# Clinical characteristics and outcomes of primary versus secondary gastrointestinal mantle cell lymphoma

**DOI:** 10.1038/s41408-020-00394-z

**Published:** 2021-01-07

**Authors:** Alessia Castellino, Aung M. Tun, Yucai Wang, Thomas M. Habermann, Rebecca L. King, Kay M. Ristow, James R. Cerhan, David J. Inwards, Jonas Paludo, Stephen M. Ansell, Thomas E. Witzig, Grzegorz S. Nowakowski

**Affiliations:** 1 Division of Hematology, Mayo Clinic, Rochester, MN USA; 2 Department of Hematology, Santa Croce e Carle Hospital, Cuneo, Italy; 3 Division of Hematologic Malignancies and Cellular Therapeutics, The University of Kansas, Kansas City, KS USA; 4 Division of Hematopathology, Mayo Clinic, Rochester, MN USA; 5 Department of Health Sciences Research, Mayo Clinic, Rochester, MN USA

**Keywords:** B-cell lymphoma, B-cell lymphoma

## Abstract

Primary gastrointestinal (GI) mantle cell lymphoma (MCL) is rare and the optimal management is unknown. We reviewed 800 newly diagnosed MCL cases and found 22 primary (2.8%) and 79 (9.9%) secondary GI MCL cases. Age, sex, and performance status were similar between primary and secondary cases. Secondary cases had more elevations in lactate dehydrogenase (28% vs 0%, *P* = 0.03) and a trend for a higher MCL international prognostic index (*P* = 0.07). Observation or local therapy was more common for primary GI MCL (29% vs 8%, *P* < 0.01), and autologous stem-cell transplant was more common for secondary GI MCL (35% vs 14%, *P* < 0.05). The median follow-up was 85 months. Primary and secondary GI MCL had similar 5-year progression-free survival (PFS) (30% vs 28%, *P* = 0.59) and overall survival (OS) (65% vs 66%, *P* = 0.83). The extent of GI involvement in primary GI MCL affected treatment selection but not outcome, with a 5-year PFS of 43% vs 14% vs 31% (*P* = 0.48) and OS of 57% vs 71% vs 69% (*P* = 0.54) in cases with single lesion vs multiple lesions in 1 organ vs multiple lesions in ≥2 organs. Less aggressive frontline treatment for primary GI MCL is reasonable. It is unknown whether more aggressive treatment can result in improved outcomes.

## Highlights

- Primary GI MCL is rare. These patients tend to be treated less aggressively in the frontline setting yet have similar outcomes as those with secondary GI MCL.
- Less aggressive frontline treatment for primary GI MCL is reasonable given decent outcome, although it is unknown whether more aggressive treatment can result in improved outcomes.

## Introduction

Mantle cell lymphoma (MCL) is a distinct subtype of mature B-cell neoplasm constituting approximately 3–10% of non-Hodgkin Lymphomas^1–6^. It is most commonly diagnosed in older males with the median age of onset approximately 68 years^4,5^. The t(11;14)(q13;q32) translocation involving *IGH* and *CCND1* genes is generally considered to be a primary genetic event leading to cyclin D1 overexpression and cell cycle dysregulation^7,8^. Recent studies suggest heterogeneity of MCL with a conventional, more aggressive nodal type and a less common, indolent type with primarily leukemic and splenic involvement^9^. Most patients present with advanced stage disease, and the disease may involve lymph nodes, spleen, as well as extranodal sites^7^. Secondary gastrointestinal (GI) tract involvement in patients with MCL (involving nodal and/or other extranodal tissue) is considered to be common and can be detected at the time of diagnosis and/or relapse. The reported prevalence was 15–30% in several retrospective studies^10–12^. However, when routine endoscopies were performed in patients with untreated MCL, GI involvement was detected in up to 90% of patients, although most patients showed no GI symptoms^13,14^. In contrast, primary GI MCL (without additional nodal or extranodal involvement) is a rare presentation that represents only 4–9% of all primary GI non-Hodgkin lymphomas^15,16^. Primary GI MCL was initially described as multiple lymphomatous polyposis^17^. It can also present as a single polyp, submucosal masses, ulcerative lesions, or diffuse mucosal infiltration^13,17–20^.

As primary GI MCL is uncommon and has significant heterogeneity in clinical presentation, the optimal management remains unclear. In addition, how primary GI MCL compares to secondary GI MCL (i.e., primary nodal MCL with concurrent, biopsy-proven GI involvement at diagnosis) in terms of clinical presentation and treatment outcome has not been well studied. Although a number of case reports are available in the literature, other studies on primary GI MCL are mainly small series with less than 10 cases reporting different outcomes^21,22^. The 5 cases of primary GI MCL with non-blastoid morphology in the British Columbia cohort were observed initially and treated at a later time when indicated, and these patients (presumably with indolent diseases) had a good outcome with a median overall survival (OS) not reached after a follow-up of 4–5 years^21^. In contrast, the 7 patients in another series did poorly overall, with only 1 of 5 patients achieving complete remission after cyclophosphamide, doxorubicin, vincristine, and prednisone (CHOP) chemotherapy while the other 4 had inadequate responses and a median OS of 6 months, although the majority of these patients had blastoid or diffuse variants^22^. An earlier study reported 31 cases of multiple lymphomatous polyposis, but true primary GI MCL cases, as defined per Dawson criteria^23^, were less than 10 in that series^17^. The European MCL Network did the retrospective study of 127 patients with isolated or predominant extranodal MCL that also included 32 patients with primary GI presentation. The median OS of 9.8 years was achieved in the overall study population, but the outcomes of isolated GI cases were not separately reported^24^.

In this study, we sought to compare the clinical characteristics including sites of GI involvement and types of GI lesions, the treatment patterns, and the survival outcomes of patients with primary vs secondary GI MCL, and to investigate whether extent of GI involvement impacted treatment selection and outcome in patients with primary GI MCL.

## Methods

### Patient selections

The Mayo Clinic institutional review board reviewed and approved the study. Adult patients (age > 18) with biopsy-proven GI involvement of MCL diagnosed between January 1, 1990 and February 28, 2018 were identified from the Mayo Clinic Lymphoma Clinical Database^25,26^. Pathology was reviewed and confirmed by expert pathologists at Mayo Clinic. The Mayo component of the Molecular Epidemiology Resource (MER) of the University of Iowa/Mayo Clinic Lymphoma Specialized Program of Research Excellence (SPORE)^27^, which was established in September 2002, was cross-referenced for additional patients with GI involvement of MCL. Demographic, clinical, and pathological characteristics, treatment information, and follow-up information were abstracted from both databases. Additional chart review was conducted to extract information on GI symptoms, GI involvement pattern, and to verify treatment and follow-up information.

Primary GI MCL was defined as per Dawson criteria^23^ which include (1) absence of palpable superficial lymph nodes; (2) no chest radiographic evidence of enlarged mediastinal lymph nodes; (3) normal total and differential white blood cell (WBC) count; (4) predominant involvement of GI tract with no lymphatic involvement beyond regional lymph nodes; and (5) no evidence of liver and spleen involvement. Patients with elevated WBC count due to neutrophilia but meeting all other Dawson criteria were deemed to have primary GI MCL. The original criterion (4) was based on laparotomy which is not incorporated into routine clinical practice. Therefore, for this study, patients who met Dawson criteria but had obvious non-regional lymph node involvement on imaging (e.g., CT or PET/CT), or biopsy-confirmed bone marrow involvement, were assigned to secondary GI MCL group. Secondary GI MCL was defined as MCL that concurrently involves the GI tract at diagnosis as well as other sites such as non-regional lymph node, liver, spleen, bone marrow, or any other extranodal sites.

Frontline therapeutic strategies were classified into five different categories: (1) observation or localized treatment with surgery or radiation; (2) less aggressive therapy, such as rituximab (R)-CHOP, R-bendamustine, R-cladribine, R monotherapy, and chemotherapy with CHOP or cladribine, without autologous stem-cell transplant (ASCT); (3) less aggressive therapy followed by ASCT; (4) aggressive therapy, such as with dose-intensified immunochemotherapy with R-maxi-CHOP alternating with rituximab and high-dose cytarabine, or R-CHOP alternating with R-DHAP (dexamethasone, cytarabine, and cisplatin), R plus hyperfractionated cyclophosphamide, vincristine, doxorubicin, and dexamethasone alternating with R plus high-dose methotrexate and cytarabine (R-HyperCVAD), without ASCT; and (5) aggressive therapy followed by ASCT. Treatment response was evaluated by treating physicians and was abstracted from the databases and/or chart review when necessary.

### Statistical analyses

Baseline clinical and pathological characteristics and therapeutic categories between patients with primary GI MCL and secondary GI MCL were compared using Chi-square or Fisher’s exact test. Progression-free survival (PFS) was defined as the time from diagnosis to disease progression or relapse, unplanned retreatment after frontline regimen, or death from any cause. OS was defined as the time from diagnosis to death from any cause. Time to event data (PFS and OS) were analyzed using the Kaplan-Meier method and the Cox proportional hazards model. All statistical analyses were performed using SPSS version 25 (IBM). A *P* value < 0.05 was considered statistically significant.

## Results

### Prevalence and clinical characteristics of primary GI MCL

A total of 800 patients with MCL were identified from the Mayo Clinic Lymphoma Database and the MER. Twenty-two (2.8%) patients presented with primary GI MCL while 79 (9.9%) presented with secondary GI MCL at diagnosis, and all cases had a biopsy-proven histopathologic evidence of GI involvement (Supplemental Fig. 1). Baseline characteristics of primary GI MCL cases are summarized in Tables 1 and 2. The median age at diagnosis was 62 years (range 43–79). There was a substantial male preponderance with a male to female ratio of 19:3. Of the primary GI MCL patients, 95% (18/19) had an Eastern Cooperative Oncology Group (ECOG) performance status of <2. Three (14%) patients had B symptoms. No patient had an elevated lactate dehydrogenase (LDH). GI symptoms leading to the diagnosis were recorded in 16 (73%) patients, with abdominal pain (*n* = 7, 44%) and GI bleeding (*n* = 5, 31%) being the most common presentations. The other six (27%) patients were diagnosed incidentally on routine screening colonoscopy. The GI presentation was heterogeneous and was detected as single lesion (mucosal, polyp, or mass; *n* = 7, 32%), multiple lesions confined to 1 organ (*n* = 7, 32%), and multiple lesions in ≥2 organs (*n* = 8, 36%). The sites of MCL involvement included colon (*n* = 15, 68%), small bowel (*n* = 9, 41%), stomach (*n* = 7, 32%), rectum (*n* = 2, 9%) and appendix (*n* = 1, 5%).

**Table 1:** Baseline characteristics of patients with primary and secondary GI MCL.

	Primary GI MCL, *n*	%	Secondary GI MCL, *n*	%	*P* value
Age					0.53
≤60 y	10	45.5	30	38.0	
>60 y	12	54.5	49	62.0	
Sex					0.75
Male	19	86.4	66	83.5	
Female	3	13.6	13	16.5	
ECOG performance status					0.68^a^
<2	18	94.7	67	89.3	
≥2	1	5.3	8	10.7	
Missing	3		4		
B symptoms					0.76^a^
Yes	3	14.3	15	19.2	
No	18	85.7	63	80.8	
Missing	1		1		
Sites of GI involvement					0.43
Upper GI trac	4	18.2	7	8.9	
Lower GI tract	14	63.6	59	74.7	
Both upper and lower GI tract	4	18.2	13	16.5	
GI lesion type					0.12
Single polyp	3	13.6	13	17.3	
Multiple polyps	14	63.6	25	33.3	
Mass lesions	2	9.1	16	21.3	
Ulceration, erosion, or nodular lesion	3	13.6	17	22.7	
Random biopsy	0	0	4	5.3	
Missing	0		4		
Histologic subtype					1.00^a^
Classic MCL	21	95.5	73	94.8	
Blastoid variant	1	4.5	4	5.2	
Missing	0		2		
LDH					0.03^a^
Normal	14	100.0	51	71.8	
Elevated	0		20	28.2	
Missing	8	0.0	8		
WBC count					0.33^a^
Normal	12	85.7	51	70.8	
Elevated	2	14.3	21	29.2	
Missing	8		7		
MIPI risk					0.07
Low	10	71.4	29	40.8	
Intermediate	1	7.1	25	35.2	
High	3	21.4	17	23.9	
Missing	8		8		

^a^Fisher’s exact test. Others were Chi-square test.

*Abbreviations*: *GI* gastrointestinal, *MCL* mantle cell lymphoma, *ECOG PS* Eastern Cooperative Oncology Group performance status, *LDH* lactate dehydrogenase, *WBC* white blood cell, *MIPI* MCL international prognostic index.

**Table 2:** Summary of the 22 primary GI MCL cases.

Case #	Age/sex	GI presentation	Year of Dx	Initial therapy	Response	Progression	PFS (mo)	Subsequent therapy	Vital status, COD	OS (mo)
1	56/M	Multiple polyps in small bowel	1992	Unknown	Unknown	Yes	6.7	ASCT	D, MDS	92.5
2	70/M	Multiple polyps in colon and rectum	1994	Surgery followed by observation	CR	Yes	72.9	Missing	D, Unknown	224.6
3	68/M	Multiple polyps in colon	1996	CHOP	CR	Yes	25.5	Cladribine	D, MCL	49.3
4	78/M	Multiple polyps in colon	1997	Radiation therapy followed by observation	CR	Yes	28.5	Rituximab	A	215.2
5	55/M	Multiple polyps in colon	1997	CHOP	CR	Yes	35.3	Cladribine	A	101.5
6	77/F	Single polyp in terminal ileum	2000	Polypectomy followed by observation	CR	Unknown	118.0	Unknown	D, Unknown	118.0
7	45/M	Multiple polyps in colon	2001	CHOP	PR	Unknown	42.7	Unknown	D, Unknown	42.7
8	45/M	Multiple polyps in stomach, small bowel, colon, and appendix	2001	R-CHOP followed by ASCT	CR	Yes	48.7	Allogeneic stem-cell transplant	A	194.5
9	55/M	Multiple polyps in stomach, small bowel, and colon	2002	R-HyperCVAD	CR	Yes	37.1	Rituximab and then gemcitabine	D, MCL	81.3
10	50/M	Multiple polyps in duodenum, small bowel, and colon	2002	R-CHOP	CR	Yes	32.5	Cladribine and then bortezomib	D, MCL	59.9
11	47/M	Areas of mild nodularity in the stomach	2003	*Helicobacter pylori* therapy and observation	CR	Yes	18.8	R-CHOP followed by ASCT	A	80.3
12	69/M	Single polyp in colon	2003	Rituximab monotherapy	CR	Yes	84.3	Rituximab and then everolimus	D, others	94.6
13	43/M	A large ulcerated gastric mass with regional lymphadenopathy	2004	R-CHOP followed by ibritumomab tiuxetan	CR	Yes	9.0	High-dose chemotherapy followed by ASCT	D, MCL	35.0
14	62/M	A colonic mass and an ileal mass with regional lymphadenopathy	2006	R-CHOP	CR	Yes	81.8	Cladribine	A	84.3
15	79/M	Multiple polyps in colon	2007	Rituximab monotherapy	CR	Yes	17.3	Rituximab monotherapy	A	141.9
16	49/M	Multiple polyps in colon and small bowel	2008	R-maxi CHOP alternating with R-AraC followed by ASCT	CR	No	33.2	Not applicable	A	33.2
17	73/M	Single polyp in colon	2009	Polypectomy followed by observation	CR	Yes	95.5	Bendamustine and rituximab	A	102.1
18	73/F	Single gastric lesion	2009	Bendamustine and rituximab	CR	No	27.8	Not applicable	D, Pancreatic cancer	27.8
19	79/F	Single polyp in stomach and multiple lesions in terminal ileum, cecum, and colon	2010	Bortezomib-containing regimen	Unknown	Yes	11.2	Unknown	D, Unknown	19.3
20	62/M	Multiple polyps in rectum and small bowel	2011	R-HyperCVAD	PD	Yes	3.5	Bendamustine and rituximab	A	8.2
21	51/M	Multiple polyps in colon	2012	Bendamustine and rituximab followed by ASCT	CR	No	67.5	Not applicable	A	67.5
22	61/M	Patchy areas of erythema and erosion in stomach	2014	Observation	SD	Yes	15.7	Bendamustine and rituximab followed by rituximab maintenance	D, Unknown	48.4

*Abbreviations*: *GI* gastrointestinal, *MCL* mantle cell lymphoma, *M* male, *F* female, *Dx* diagnosis, *CHOP* cyclophosphamide, doxorubicin, vincristine, prednisone, *R* rituximab, *R-HyperCVAD* rituximab plus hyperfractionated cyclophosphamide, vincristine, doxorubicin, and dexamethasone alternating with rituximab plus high-dose methotrexate and cytarabine, *ASCT* autologous stem-cell transplant, *CR* complete response, *PR* partial response, *SD* stable disease, *PD* progressive disease, *PFS* progression-free survival, *OS* overall survival, *A* alive, *D* deceased, *COD* cause of death, *mo* months MDS myelodysplastic syndrome.

### Clinicopathological characteristics of primary vs secondary GI MCL

There were no statistically significant differences identified between patients with primary and secondary GI MCL with regard to age (≤60 vs >60), sex (male vs female), ECOG performance status (<2 vs ≥2), or B symptoms (Table 1). The distribution patterns of site of GI involvement (upper GI tract vs lower GI tract), GI lesion type, and histology (classic vs blastoid) were similar between patients with primary and secondary GI MCL. Compared with patients with secondary GI MCL, patients with primary GI MCL had fewer elevations in LDH (*P* = 0.03). There was a trend of lower MCL international prognostic index (MIPI) score in patients with primary GI MCL (*P* = 0.07).

### Treatment pattern of primary vs secondary GI MCL

The treatment patterns for patients with primary and secondary GI MCL are summarized in Table 3. More patients with primary GI MCL were initially observed or treated with localized therapy (28.6% vs 7.6%, *P* < 0.01), while more patients with secondary GI MCL underwent ASCT as part of frontline treatment (35.4% vs 13.6%, *P* < 0.05).

**Table 3:** Treatment pattern in patients with primary and secondary GI MCL.

	Primary GI MCL		Secondary GI MCL	
	*n*	%	*n*	%
Frontline therapy				
Observation and/or localized therapy	6	28.6	6	7.6
Less aggressive therapy without ASCT	10	47.6	40	50.6
Less aggressive therapy with ASCT	2	9.5	17	21.5
Aggressive therapy without ASCT	2	9.5	5	6.3
Aggressive therapy with ASCT	1	4.8	11	13.9
Missing	1			

	Single lesion		Multiple lesions in 1 organ		Multiple lesions in ≥2 organs	
	*n*	%	*n*	%	*n*	%
Frontline therapy						
Observation and/or localized therapy	4	57.1	1	16.7	1	12.5
Less aggressive therapy without ASCT	3	42.9	4	66.7	3	37.5
Less aggressive therapy with ASCT	0	0	1	16.7	1	12.5
Aggressive therapy without ASCT	0	0	0	0	2	25.0
Aggressive therapy with ASCT	0	0	0	0	1	12.5
Missing			1			

*Abbreviations*: *GI* gastrointestinal, *MCL* mantle cell lymphoma, *ASCT* autologous stem-cell transplant.

For patients with primary GI MCL, there was a trend of more aggressive therapy in patients with more extensive GI involvement. Seven patients presented with a single lesion; among those, 2 were initially observed, 2 underwent polypectomy only, and 3 were treated with systemic therapy (1 with rituximab alone, 1 with R-CHOP, and 1 with R-bendamustine). Seven patients presented with multiple lesions in 1 organ; among those, 1 underwent radiotherapy alone, 4 were treated with systemic therapy (1 with rituximab alone, 3 with CHOP), 1 was treated with R-bendamustine followed by ASCT, and treatment was unclear for 1 patient. Eight patients presented with multiple lesions involving multiple organs; among those, 1 underwent surgical resection only (multiple polyps in the colon and the rectum), 2 were treated with R-CHOP, 1 was treated with bortezomib-containing regimen, 2 were treated with R-HyperCVAD, and 2 were treated with chemotherapy (1 with CHOP and 1 with Nordic regimen) followed by ASCT.

### Outcomes of primary vs secondary GI MCL

**Fig. 1:**
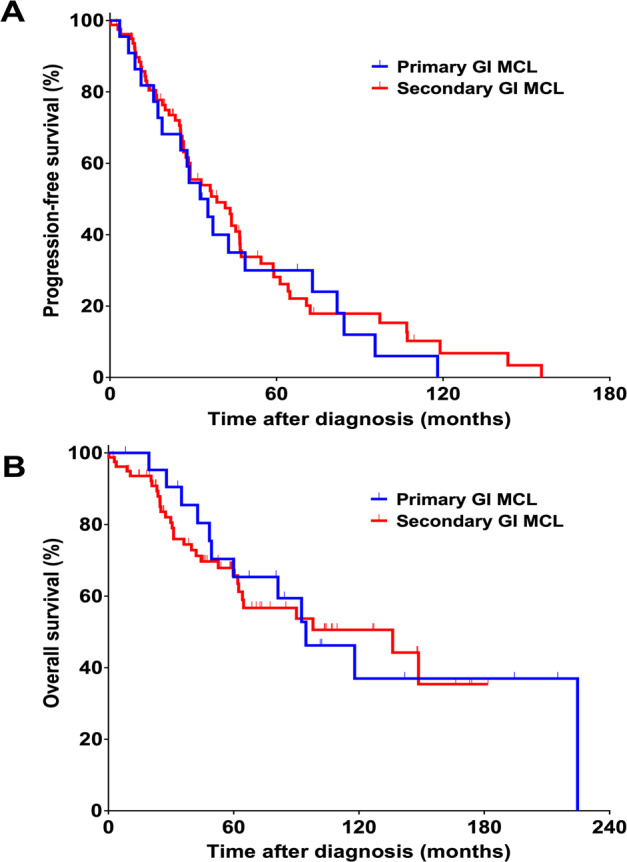
PFS and OS of patients with primary vs secondary GI MCL. Kaplan-Meier curves of PFS (A) and OS (B) of patients with primary (*n* = 22) vs secondary (*n* = 79) GI MCL. Abbreviations: PFS progression-free survival, OS overall survival, GI gastrointestinal, MCL mantle cell lymphoma.progression-free survival, *OS* overall survival, *GI* gastrointestinal, *MCL* mantle cell lymphoma.

The median follow-up time for the entire cohort was 85.0 months (95% CI 53.4–116.6). The median PFS was 32.5 months (95% CI 21.4–43.6) for patients with primary GI MCL and 38.5 months (95% CI 22.9–54.2) for patients with secondary GI MCL (Fig. 1a). The 5-year PFS rates were 30.0% and 28.1%, respectively (*P* = 0.59). The median OS was 94.6 months (95% CI 56.2–133.0) for patients with primary GI MCL and 136.1 months (95% CI 39.3–233.0) for patients with secondary GI MCL (Fig. 1b). The 5-year OS rates were 65.3% and 65.8%, respectively (*P* = 0.83). In the Cox regression model, after adjusting for LDH, patients with primary and secondary GI MCL had similar PFS (HR 1.32, 95% CI 0.67–2.57, *P* = 0.42) and OS (HR 0.94, 95% CI 0.37–2.42, *P* = 0.90).

### Outcomes of primary GI MCL by extent of GI involvement

**Fig. 2:**
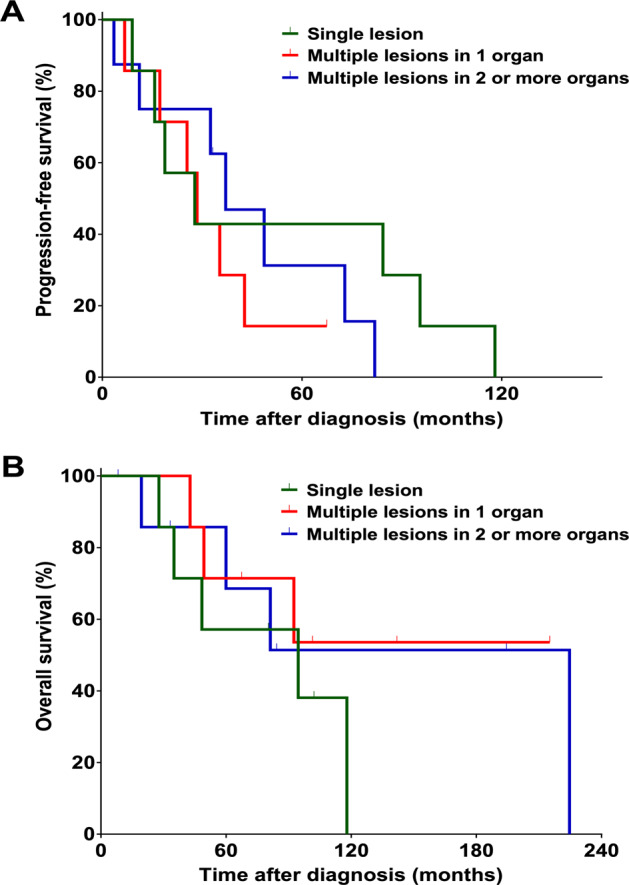
PFS and OS of patients with primary GI MCL with different extent of GI involvement. Kaplan-Meier curves of PFS (A) and OS (B) of primary GI MCL cases with single lesion (*n* = 7) vs multiple lesions in 1 organ (*n* = 7) vs multiple lesions in ≥2 organs (*n* = 8). Abbreviations: PFS progression-free survival, OS overall survival, GI gastrointestinal, MCL mantle cell lymphoma.*Abbreviations*: *PFS* progression-free survival, *OS* overall survival *GI* gastrointestinal, MCL mantle cell lymphoma.

In patients with primary GI MCL, the median PFS was 27.8 months (95% CI 4.8–50.7) for patients with a single lesion, 28.5 months (95% CI 20.7–36.3) for patients with multiple lesions in 1 organ, and 37.1 months (95% CI 18.2–56.0) for patients with multiple lesions in ≥2 organs (Fig. 2a). The 5-year PFS rates were 42.9%, 14.3%, and 31.3%, respectively (*P* = 0.48). The median OS was 94.6 months (95% CI 27.8–NA) for patients with a single lesion, not reached (95% CI 42.7–NA) for patients with multiple lesions in 1 organ, and 224.6 months (95% CI 19.3–NA) for patients with multiple lesions in ≥2 organs (Fig. 2b). The 5-year OS rates were 57.1%, 71.4%, and 68.6%, respectively (*P* = 0.54).

## Discussion

To our knowledge, our study represents one of the largest series of GI MCL that have been reported. We found that primary GI MCL was uncommon (less than 3% of all MCL), had a heterogeneous clinical presentation, and had similar outcomes compared to secondary GI MCL. Primary GI MCL is considered to be a rare clinical presentation, and it accounted for 4–9% of all primary GI non-Hodgkin lymphomas^15,16^. The Dawson criteria^23^ of primary GI lymphoma was reported in 1961. There have since been significant advances in imaging and laboratory analysis, namely PET/CT and flow cytometry. Therefore, the diagnosis of primary GI lymphoma in modern practice does not need to follow the Dawson criteria. Practically, a diagnosis of primary GI lymphoma can be made if there is no additional nodal or extranodal involvement of lymphoma in a case of documented GI lymphoma. In the case of primary GI MCL, there can be no nodal, extranodal (including bone marrow), or peripheral blood involvement of MCL. With these criteria, primary GI MCL accounted for 2.8% of all MCL cases, confirming that this presentation is rare. This result is similar to data reported in the literature. In a previous cohort reported from British Columbia, primary GI MCL accounted for 1.1% (5/440) of the MCL cases^21^. In contrast, secondary GI MCL accounted for approximately 10% of the MCL cases in our study, somewhat lower than data reported in the literature (15–30%)^10–12^, likely due to different study populations, the study era, and different incorporation of endoscopy (i.e., routine endoscopy for staging was not universally done in this cohort).

We found that patients with primary GI MCL cases tended to have lower MIPI scores compared to secondary GI MCL cases. The findings are consistent with a lower disease burden in patients with primary GI MCL. These patients with primary GI MCL were treated less aggressively, with more patients observed or receiving local therapy alone, and fewer patients undergoing ASCT in those treated with systemic therapy. However, the treatment outcomes were similar between patients with primary and secondary GI MCL. This suggests that in patients with MCL in the GI tract only, it is reasonable to treat less aggressively, e.g., observation when appropriate, local therapy when feasible, and consider less intensive chemotherapy if systemic therapy is indicated. On the other hand, it is unknown whether more aggressive therapy in patients with primary GI MCL can result in better outcomes. It is not known whether primary and secondary GI MCL have similar biology that dictates the similar outcome. Molecular studies of the GI lymphoma sample in these cases may provide valuable insights.

The extent of GI involvement appeared to have impacted the treatment strategy in our series of patients with primary GI MCL. For instance, patients with a single lesion were more likely to be observed or treated with surgery or radiation alone, while patients with multiple lesions (involving 1 or 2+ organs) were more likely to receive intensive systemic therapy and ASCT. The treatment outcomes were similar though in patients with different extent of GI involvement. This suggests that in patients with a lower disease burden, less aggressive initial treatment may be reasonable. For example, for patients with a single GI lesion, local therapy, if feasible, may be appropriate. However, we have to note that relapses are still common in patients presenting with one single lesion (as shown in Fig. 2 and detailed in Table 2). Notably, in patients with MCL, GI involvement was found in up to 90% of the patients when routine endoscopies were done^13,14^. The majority of them were detected by random biopsies of endoscopically normal mucosa^13,14^. Thus, most patients with a single GI lesion may well have multifocal diseases that can result in clinical relapses if no systemic treatment was given initially. In patients with lower burden of GI MCL, e.g., single lesion, while limited therapy at diagnosis and reserving more aggressive systemic treatment for relapse is certainly reasonable and results in decent outcome, it is unknown whether upfront, more aggressive systemic treatment would improve long-term outcome.

The strengths of our study include a systematic review of consecutive MCL cases in the Mayo Clinical Lymphoma Database for identification of GI involvement, a large cohort size of MCL patients, a significant number of primary GI MCL cases for a rare disease, a comprehensive comparison of primary vs secondary GI MCL, as well as closer investigation of the impact of extent of GI involvement which provides potentially important clinical practice implications. The weaknesses of the study include a retrospective design (prospective follow-up only in a subset of patients who were enrolled in MER), a long study period (January 1, 1990–February 28, 2018) that spans different eras of MCL management with potentially different staging and treatment modalities, diverse treatment strategies and regimens over the years, and missing information in some patients who did not follow-up consistently at our institution. Only limited number of patients in this cohort had the opportunity to receive modern treatments such as immunomodulatory drug (IMiD) such as lenalidomide, Bruton’s tyrosine kinase (BTK) inhibitors such as ibrutinib, BCL-2 inhibitor such as venetoclax^28–35^. These efficacious novel therapies, as well as the most recently approved CD19 chimeric antigen receptor (CAR) T-cell therapy, may improve the outcomes of both primary and secondary GI MCL^36^.

In summary, primary GI MCL is a rare and distinct presentation. It tends to be treated less aggressively, especially in patients with a lesser extent of GI involvement, but has a similar outcome to that of secondary GI MCL. Therefore, less aggressive treatment of primary GI MCL is reasonable. Whether an intensified systematic treatment approach for primary GI MCL can result in better outcome is unknown. A better understanding of the molecular biology is needed, and the incorporation of novel therapeutic agents should be explored for patients with primary GI MCL.

## Supplementary information

Supplemental Figure 1.

Reproducibility checklist

## References

[1] Teras LR (2016). 2016 US lymphoid malignancy statistics by World Health Organization subtypes. CA Cancer J. Clin..

[2] Al-Hamadani M (2015). Non-Hodgkin lymphoma subtype distribution, geodemographic patterns, and survival in the US: a longitudinal analysis of the National Cancer Data Base from 1998 to 2011. Am. J. Hematol..

[3] Shiels MS (2013). The epidemic of non-Hodgkin lymphoma in the United States: disentangling the effect of HIV, 1992–2009. Cancer Epidemiol. Biomarkers Prev..

[4] Fu S (2017). Trends and variations in mantle cell lymphoma incidence from 1995 to 2013: a comparative study between Texas and National SEER areas. Oncotarget.

[5] Cheah CY, Seymour JF, Wang ML (2016). Mantle cell lymphoma.. J. Clin. Oncol..

[6] Jain P, Wang M (2019). Mantle cell lymphoma: 2019 update on the diagnosis, pathogenesis, prognostication, and management. Am. J. Hematol..

[7] Inwards DJ, Witzig TE (2011). Initial therapy of mantle cell lymphoma. Ther. Adv. Hematol..

[8] Li JY (1999). Detection of translocation t(11;14)(q13;q32) in mantle cell lymphoma by fluorescence in situ hybridization. Am. J. Pathol..

[9] Swerdlow SH (2016). The 2016 revision of the World Health Organization classification of lymphoid neoplasms. Blood.

[10] Samaha H (1998). Mantle cell lymphoma: a retrospective study of 121 cases. Leukemia.

[11] Chim CS (1998). Mantle cell lymphoma in the Chinese: clinicopathological features and treatment outcome. Am. J. Hematol..

[12] Yatabe Y (2000). Significance of cyclin D1 overexpression for the diagnosis of mantle cell lymphoma: a clinicopathologic comparison of cyclin D1-positive MCL and cyclin D1-negative MCL-like B-cell lymphoma. Blood.

[13] Romaguera JE (2003). Frequency of gastrointestinal involvement and its clinical significance in mantle cell lymphoma. Cancer.

[14] Salar A (2006). Gastrointestinal involvement in mantle cell lymphoma: a prospective clinic, endoscopic, and pathologic study. Am. J. Surg. Pathol..

[15] Ruskoné-Fourmestraux A, Audouin J (2010). Primary gastrointestinal tract mantle cell lymphoma as multiple lymphomatous polyposis. Best Pract. Res. Clin. Gastroenterol..

[16] Kohno S (2003). Clinicopathological analysis of 143 primary malignant lymphomas in the small and large intestines based on the new WHO classification. Histopathology.

[17] Ruskone-Fourmestraux A (1997). Multiple lymphomatous polyposis of the gastrointestinal tract: prospective clinicopathologic study of 31 cases. Gastroenterology.

[18] Cornes JS (1961). Multiple lymphomatous polyposis of the gastrointestinal tract. Cancer.

[19] Fraga M (1995). Mucosal mantle cell (centrocytic) lymphomas. Histopathology.

[20] Bairey O, Ruchlemer R, Shpilberg O (2006). Non-Hodgkin’s lymphomas of the colon. Isr. Med. Assoc. J..

[21] Abrisqueta P (2017). Observation as the initial management strategy in patients with mantle cell lymphoma. Ann. Oncol..

[22] Dasappa L (2014). Primary gastrointestinal mantle cell lymphoma: a retrospective study. J. Gastrointest. Cancer.

[23] Dawson IMP, Cornes JS, Morson BC (1961). Primary malignant lymphoid tumours of the intestinal tract. Report of 37 cases with a study of factors influencing prognosis. Br. J. Surg..

[24] Morello L (2019). Mantle cell lymphoma of mucosa-associated lymphoid tissue: a European mantle cell lymphoma network study. HemaSphere.

[25] Castellino A (2019). Role of systemic high-dose methotrexate and combined approaches in the management of vitreoretinal lymphoma: a single center experience 1990-2018. Am. J. Hematol..

[26] St-Pierre F (2019). Detection of extranodal and spleen involvement by FDG-PET imaging predicts adverse survival in untreated follicular lymphoma. Am. J. Hematol..

[27] Cerhan JR (2017). Cohort profile: the Lymphoma Specialized Program of Research Excellence (SPORE) Molecular Epidemiology Resource (MER) Cohort Study. Int. J. Epidemiol..

[28] Wang ML (2013). Targeting BTK with ibrutinib in relapsed or refractory mantle-cell lymphoma. N. Engl. J. Med..

[29] Wang M (2018). Acalabrutinib in relapsed or refractory mantle cell lymphoma (ACE-LY-004): a single-arm, multicentre, phase 2 trial. Lancet.

[30] Song Y (2018). Safety and activity of the investigational Bruton tyrosine kinase inhibitor zanubrutinib (BGB-3111) in patients with mantle cell lymphoma from a phase 2 trial. Blood.

[31] Tam CS (2018). Ibrutinib plus venetoclax for the treatment of mantle-cell lymphoma. N. Engl. J. Med..

[32] Davids MS (2017). Phase I first-in-human study of venetoclax in patients with relapsed or refractory non-Hodgkin lymphoma. J. Clin. Oncol..

[33] Eyre TA (2019). Efficacy of venetoclax monotherapy in patients with relapsed, refractory mantle cell lymphoma after Bruton tyrosine kinase inhibitor therapy. Haematologica.

[34] Tun AM, Ansell SM (2020). Immunotherapy in Hodgkin and non-Hodgkin lymphoma: Innate, adaptive and targeted immunological strategies. Cancer Treat. Rev..

[35] Goy A (2012). Phase II multicenter study of single-agent lenalidomide in subjects with mantle cell lymphoma who relapsed or progressed after or were refractory to bortezomib: the MCL-001 “EMERGE” Study. Blood.

[36] Wang M (2020). KTE-X19 CAR T-cell therapy in relapsed or refractory mantle-cell lymphoma. N. Engl. J. Med..

