# Supplementary material for: Clinical characteristics and outcomes of primary versus secondary gastrointestinal mantle cell lymphoma
Source: Blood Cancer J. 2021 Jan 7;11(1):8. doi: 10.1038/s41408-020-00394-z (PMC7791108; doi:10.1038/s41408-020-00394-z)
Supplement: Supplementary file 1 — Supplemental Figure 1. [file 41408_2020_394_MOESM1_ESM.pdf]

Supplemental Figure 1. Description of study patient selection

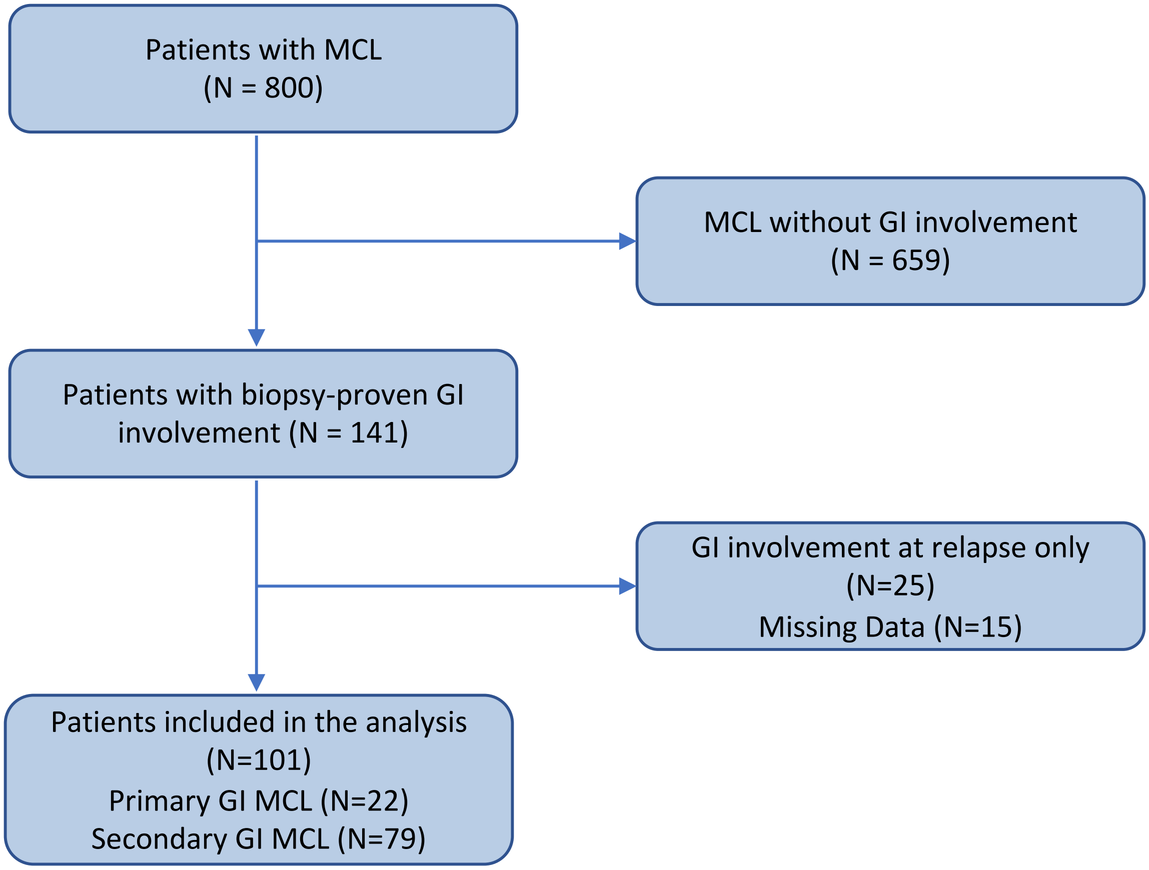

Abbreviations used: MCL, mantle cell lymphoma; GI, gastrointestinal.
